# Electroencephalography (EEG) Evidence for the Psychological Processes of Humor Generation: A Comparison Perspective on Humor and Creativity

**DOI:** 10.3390/bs14040290

**Published:** 2024-03-31

**Authors:** Cuicui Sun, Zhijin Zhou

**Affiliations:** 1 Sichuan Key Laboratory of Psychology and Behavior of Discipline Inspection and Supervision, School of Psychology, Sichuan Normal University, Chengdu 610068, China; suncc@sicnu.edu.cn; 2 School of Psychology, Central China Normal University, Wuhan 430079, China

**Keywords:** humor generation, creative generation, humor dialogue, alpha power, EEG

## Abstract

(1) Background: Humor stands out as the most dynamic and innovative aspect of human intelligence. Drawing on the cognitive parallels between humor and creativity, this study explored the EEG alpha frequency band activity patterns during humor generation by comparing the process of generating humorous and creative ideas. (2) Methods: Thirty-six participants were randomly assigned to either the humor generation group or the creative generation group, and the dependent variable was the neural oscillation in both low-frequency and high-frequency alpha during the early, middle, and late stages of both humor and creative generation. (3) Results: In the early stages, both humor and creative generation exhibited significantly higher power in low-frequency alpha and high-frequency alpha in the temporal region compared to the middle and late stages. In the middle and late stages, the low-frequency alpha oscillation in the frontal region for humor generation was significantly higher than that for creative generation. (4) Conclusions: Humor and creative generation share similar neural activation patterns in the early stages, involving the activation and retrieval of long-term memory information based on contextual cues. The differences between the two primarily manifest in the middle and late stages, where the selection of humorous ideas requires inhibiting not only irrelevant or ordinary ideas, akin to creative generation but also novel yet non-humorous ideas. This study sheds light on the neurocognitive mechanisms of humor generation and provides insights into the cognitive parallels and distinctions between humor generation and creative generation.

## 1. Introduction

Humor, a distinctly human expression of advanced cognitive abilities, serves as an adaptive coping mechanism, significantly shaping social interactions [1]. It spans various dimensions, including appreciation, comprehension, and production [2]. While current neuroscientific research predominantly explores the passive aspects of humor, such as comprehension and appreciation, with investigations into brain localization [3], temporal dynamics [4], and diverse groups [5,6], limited attention is given to exploring the cognitive processes and neural mechanisms associated with active humor generation. 

Researchers have characterized humor as a manifestation of spontaneous and real-life applications of creative cognition [7]. The most significant convergence of humor and creativity lies in the domain of humor generation. While appreciating or comprehending humor inherently involves a certain degree of creativity, the correlation between humor generation and creativity is more direct and robust [8]. Therefore, the present study aims to investigate the functional patterns of EEG brain activity during humor generation by comparing the shared and distinct neurocognitive mechanisms of humor generation and creative cognition. 

### 1.1. Humor Generation 

Compared with humor appreciation and comprehension—which are considered as passive processing of humorous stimuli—humor generation emphasizes individuals actively creating humor. Humor generation is defined as ‘the ability or habit of individuals to actively create new humor examples or make others laugh, as well as the internal process of humor creativity’ [9]. Ruch and Heintz suggest that humor generation comprises both humor creation and humor reproduction. Both aspects can be further categorized into quality (the degree to which humor is created or reproduced) and quantity (the frequency of humor creation or reproduction), as well as typical behavior (habit) and maximal behavior (ability) [2]. The use of different methodologies among researchers has led to discrepancies in the terminology used to describe humor generation. For instance, Amir and Biederman employed terms such as “creativity” or “creation” to represent the concept of humor generation [10]. Conversely, Feingold and Mazzella used the term “wit” to denote humor generation [11]. Moreover, even when researchers employ the same terminology, they often have different conceptual definitions of humor generation. For example, Babad and Kaufman et al. both employed the term “humor production” in their studies [12,13]. However, Babad’s interpretation emphasized the typical or habitual behavior aspect of humor generation, focusing on the invention of amusing examples [12]. In contrast, Kaufman et al. emphasized the maximizing behavior of humor generation and defined it as the ability to generate new humorous examples or amuse others [13]. 

In addition, some researchers connected humor generation with creativity and adopted the term “humor creation” to define humor generation. Humor creation was described as a kind of creativity in cognitive and social fields (creating witticisms and amusing others) [14]. Martin and Lefcourt also consider humor as a creative act that entails sudden shifts in cognitive perspectives and thinking patterns [15]. A widely accepted standard definition of creativity posits that it necessitates both originality and effectiveness [16] (p. 92). However, this definition predominantly focuses on the creative process’s achievement, neglecting considerations of inconclusiveness, time variance, and knowledge-domain dependence in evaluating the outcomes. In response to these limitations, a dynamic definition of creativity has been proposed: “Creativity requires potential originality and effectiveness” [17]. This dynamic perspective treats creativity as a fluid phenomenon, adopting a pragmatist approach that underscores the coexistence of creative achievement and inconclusiveness. Building upon these theoretical foundations, this study conceptualizes humor generation as a distinctive form of verbal creativity. In this study, humor generation is viewed as a cognitive process influenced by emotional goals, emphasizing the intricate interplay between creativity and emotion.

### 1.2. Empirical Studies of Humor Generation

#### 1.2.1. Behavioral Studies of Humor Generation

The behavioral study on humor generation generally focuses on the predictive factors of humor generation, such as personality traits and cognitive ability. Greengross et al. conducted a meta-analysis to examine the sex difference in humor generation and found that men’s humor output was rated as funnier than women’s, with a combined effect size of 0.321 [18]. Some researchers explored the effect of personality traits on humor generation and found that individuals with high openness to experience have higher verbal skills and are good at appreciating the unconventional features of humor, which is conducive to humor generation [19,20]. In addition, several researchers focused on the impact of individual domain-general ability on humor generation. It was found that intelligence and creativity can positively predict humor generation ability [8,21], and humor generation is similar to the generation of creative ideas; that is, the humor degree of the generated ideas (quality) improves with an increase in the number of ideas generated (quantity) [22]. It can be observed that researchers have provided sufficient evidence for why there are individual differences in humor generation; however, they cannot explain how individuals generate humor. It is necessary to further reveal the cognitive processes of humor generation to answer this question.

Regarding the cognitive processes involved in humor generation, Kozbelt et al. employed objective and precise reaction time measurements to assess humor generation and comprehension [23]. This approach replaced the relatively subjective self- or other-report methods and utilized signal detection models for statistical data analysis. The study revealed that during the process of humor comprehension compared with humor generation, participants exhibited shorter reaction times and greater confidence in providing correct answers. The findings support the notion that humor comprehension involves an insight characteristic, where the understanding of jokes occurs rapidly and confidently without compromising speed for accuracy. However, in the process of humor generation, there was a significant negative correlation between participants’ reaction times and their humor evaluations for each headline. This suggests that humor generation does not follow a similar insight process. The study represents an initial exploration of the cognitive processes involved in humor generation and provides valuable insights for future research. However, its focus primarily on the relationship between humor generation, insight, and humor comprehension limits its in-depth examination of the cognitive processing underlying humor generation. Sun and colleagues investigated the dynamic cognitive processes involved in humor generation, drawing parallels between humor generation and creative cognition and found that humor generation entails the cognitive processes of both activation and inhibition of information. These processes exhibit clear temporal dynamics, with activation of contextual literal information taking place initially, followed by the subsequent inhibition of literal information. The study explored the cognitive processes of humor generation based on the cognitive similarity of humor generation and creativity for the first time [9].

#### 1.2.2. Neurological Research on Humor Generation

At present, there is limited research directly investigating the neural mechanisms of humor generation. Previous extensive theoretical and empirical studies have consistently found that humor generation shares similar cognitive processes with creativity [8,9,22,24]. Hence, drawing from pertinent neural research on creative thinking or the generation of creative ideas, investigating the neural mechanisms of humor generation appears to be a viable prospect.

In previous studies on the neural mechanisms of creative thinking, researchers primarily examined the EEG power characteristics of creative tasks by comparing divergent thinking with convergent thinking or creative thinking with general thinking [25]. Despite employing diverse creative tasks and experimental paradigms, a relatively consistent conclusion has been reached that the process of generating creative ideas is often associated with an increase in alpha power, particularly in the frontal and right posterior hemisphere regions [26]. Notably, the originality of ideas is not only associated with an increase in alpha power [27,28] but also targeted interventions aimed at enhancing creativity have been shown to elevate alpha power [29]. An increase in alpha power typically denotes a decrease in cortical arousal levels and is referred to as ‘cortical idling’ [30]. The alpha effects associated with creativity can be interpreted as reflective of a low cortical arousal state, indicative of diffused attention and highly integrative thinking [31]. Gruzelier posits that the close association between creativity and alpha oscillations may arise from a potential possibility: deep relaxation states could enhance an individual’s creative potential, maximizing creative performance [32]. On the other hand, some researchers suggest that an increase in alpha power not only signifies a reduction in positive cognitive processing activity but is also closely linked to task performance associated with cognitive inhibition [33]. The synchronization of alpha activity during creative tasks may indicate a high demand for internal processing, contributing to a highly internally attentive state that facilitates the (re)combination of distant semantic information. 

While many studies have indeed identified an increase in alpha power during the creative thinking process, inconsistencies exist in the conclusions drawn by researchers. Razumnikova analyzed the changes in alpha power during convergent thinking tasks and divergent thinking tasks and found that the alpha power during both cognitive tasks was significantly lower than during rest periods [34]. Other researchers have pointed out that alpha synchronization exclusively correlates with cognitive processes in divergent thinking, as opposed to other creative thinking tasks [35]. Furthermore, distinctive variations in alpha power emerge among individuals with different levels of creative traits [36]. Specifically, those scoring high in uniqueness in AUT (alternative uses test, AUT) exhibit heightened alpha power in the right frontal and posterior regions relative to their lower-scoring counterparts. Conversely, individuals with lower creativity manifest increased alpha synchronization in the central temporal to parietal regions when generating novel ideas. In addition, although some controversy surrounds changes in alpha frequency band power during creative cognitive processes, a consensus prevails regarding the pivotal role of the frontal cortex in creative thinking, particularly in divergent thinking processes, potentially accompanied by activation in specific areas of the parietal and temporal lobes [25,36].

## 2. The Present Study

Drawing from a review of humor generation studies, the predominant focus is currently centered around an individual trait perspective. This perspective primarily explored individual factors influencing humor generation, presenting multidimensional evidence for individual variations in humor generation. However, this line of research exhibited a notable limitation—it failed to explain how individuals generate humor. To address this gap, it is imperative to delve into the cognitive processing mechanisms underlying humor generation. On the other side, research adopting a cognitive-oriented approach to humor generation is presently scant and fragmented. There is a notable absence of a direct examination of the psychological processing and neural mechanisms involved in the generation of humor. To address the shortcomings in previous research while recognizing the similarity between humor generation and creativity, this study aimed to explore the EEG alpha frequency band activity patterns during humor generation by comparing the generation of humorous and creative ideas. The primary emphasis lies in examining the fluctuations in low-frequency and high-frequency alpha power across distinct brain regions (frontal lobe, temporal lobe, and parietal lobe) during the early, middle, and late stages of generating both humorous and creative ideas. 

According to the generation-selection model of creative cognition [37], the generation and selection of humorous ideas are the key cognitive processes of humor generation. In the generation phase, individuals initially retrieve and extract task-related information from long-term memory, guided by the context of the provided humor, and generate ideas that may evoke incongruity in receivers. In the selection stage, individuals must reassess and evaluate whether the ideas generated in the earlier phase can elicit joyful emotional experiences or provoke laughter in others. Differing from creativity, where the ultimate goal is to elucidate objective facts and solve practical problems, humor generation often involves selecting viewpoints deemed absurd or even self-contradictory. This is done not for the purpose of conveying factual information but rather to convey emotions and bridge psychological distances. Hence, we hypothesized that humor generation and creative generation exhibited similar patterns of high and low-frequency alpha neural oscillations during the early stage of viewpoint generation; the distinctions were anticipated to emerge predominantly during the subsequent mid and late stages of viewpoint selection.

## 3. Methods 

### 3.1. Participants

Prior to the formal experiment, we conducted a power analysis using G*Power 3.1 to ensure the adequacy of our sample size. The significance level (alpha) was set at 0.05, and we employed an effect size of 0.25, as recommended by Cohen’s research [38]. A statistical power of 0.95 was chosen for the analysis. The results of the analysis indicated that a minimum total sample size of 12 was required.

Thirty-six undergraduate students from a university in Wuhan participated in this study. They were randomly assigned to either the humor group or the novelty group, with 18 participants in each group, aged 17–22 (*M* = 19.97, *SD* = 1.03), including 15 males and 21 females. One participant from the humor group withdrew from the experiment midway, and the data from the remaining 35 participants were included in the final statistical analysis. All participants were right-handed, healthy, had no history of mental illness or drug abuse, and possessed normal or corrected vision. Additionally, none of the participants had any prior experience with creativity or humor experiments. Before the experiment, all participants voluntarily signed written informed consent forms and were remunerated with a cash reward upon completion. The study received approval from the authorized ethics committee.

### 3.2. Experimental Tasks and Procedure

To enhance the ecological validity of the study, humor dialogue experiment materials closely linked to everyday life scenarios were employed. In each humorous dialogue, the initial sentences establish the context of the humor, while the ultimate sentence serves as the pivotal “punchline” that characterizes the humor and which, in this study, needs to be generated by the participant according to the context background provided. For example (context and background): Man A: Have you broken off the engagement with that girl? Man B: Yes, she is not willing to marry me. Man A: Didn’t you tell her that your uncle is a rich man? Man B: I did, ____. A possible humorous punchline, in this case, would be ‘so she is my aunt now’. Presently, humor dialogue-based experimental materials have been widely used in cognitive and neural mechanism studies on humor appreciation and comprehension [3,4].

The experimental materials consisted of 33 humorous dialogues compiled by Sun [39]. Thirty dialogues were utilized for the formal experiment, while three were reserved for practice. In accordance with the varying experimental tasks, participants were randomly allocated to either the humor group or the novelty group. Participants in the humor group were asked to generate an answer that could form a funny connection with the context or make people laugh, while the novelty group was asked to generate an answer that could form a novel association with the context and have general semantic rationality. Subsequently, each answer was rated on a scale of 1–7 (1 = “not humorous/novel at all” to 7 = “very humorous/novel”) by the participants themselves. The primary reason for opting for self-evaluation over external rating lies in the significant subjectivity inherent in humor. Different evaluators, influenced by their personal preferences and cultural biases, might assign considerably divergent scores to the same responses, resulting in low consistency in ratings. Utilizing self-evaluation, however, allows for direct capture of individuals’ experiences with humor and novelty, effectively sidestepping this particular concern. 

Based on self-evaluation scores, trials ranking within the top 33% of scores for humor were categorized into the high-humor group, while those ranking in the bottom 33% were classified as the low-humor group. This categorization was similarly applied to the novelty group. In light of the difficulty associated with humor and novelty generation, the data indicate that, among responses self-generated by participants, only 31.9% were self-evaluated as highly creative [40]. To delve more effectively into the distinct psychological processes underpinning humor and novelty generation, only the top 33% of responses identified as high in humor or novelty were considered for inclusion in the final statistical analysis.

**Figure 1:**
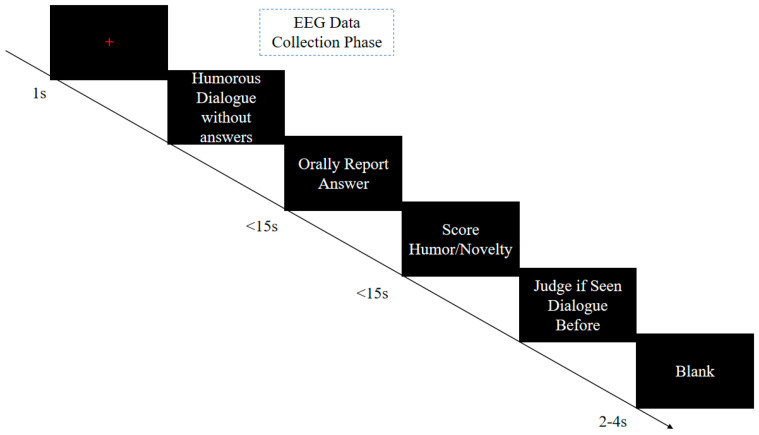
Experimental procedure.

The formal experiment procedure is illustrated in Figure 1. During the experiment, a 1000 ms “+” fixation point appears at the center of the computer screen, followed by a humorous dialogue to be completed. Participants are instructed to generate humorous or novel responses while the dialogue is presented and then press the “Enter” key to access the answer interface. Subsequently, they are allotted 15 s to vocally report their answers and rate the humor or novelty on a scale from 1 (not humorous/novel at all) to 7 (very humorous/novel). Participants are also asked to determine whether they have seen the joke previously. A blank screen, lasting 2–4 s, appears randomly between trials. To minimize the impact of artifact signals, participants are instructed to maintain stillness throughout the experiment, with two 2 min breaks interspersed. The primary focus of the formal data analysis centers on the second screen interface, specifically when participants are presented with dialogues and prompted to conceive humorous/novel answers.

### 3.3. EEG Data Acquisition and Analysis

EEG data were collected using a 64-channel stretchable electrode cap produced by Brain Products in Germany, with electrode coordinates imported using the 10-5 international coordinate system. The reference and ground electrodes were located at FCz and GND, respectively. To record eye movement, an electrode was placed under the right eye to record vertical eye electrical activity (VEOG). Impedance of the eye electrode, reference electrode, and ground electrode was kept below 10 k, while impedance of other electrodes was kept below 20 k. EEG signals were amplified and band-pass filtered between 0.05 and 100 Hz, with all signals sampled at a frequency of 500 Hz.

EEGLAB 2021.0 was used for offline data processing. The average signal of all electrodes was used to re-reference the data, and a band-pass filter of 1–50 Hz was used to remove low and high-frequency noise. Subsequently, independent component analysis (ICA) was employed to eliminate eye movement artifacts and any channel voltage exceeding ±100 μV. After data preprocessing, the first 33% of the participants’ humor or novelty scores were screened out, and the EEG signals of the first 2 s, the middle 2 s, and the last 2 s of the participants’ thinking stage were extracted to investigate the dynamic neural process of humor generation and creative cognition in the early, middle, and late time periods.

Based on prior research [25,26,27], we aim to extract the power of the low-frequency alpha band (8–10 Hz) and high-frequency alpha band (10–12 Hz) for EEG analysis. The dependence index is represented in units of μV^2^. We employ the method of creating STUDY by EEGLAB to compute the power, and subsequently, each power value is divided by 10 to obtain the value of the targeted dependent variable. The designated brain regions include the frontal lobe, temporal lobe, and parietal lobe, with corresponding electrodes as follows: in the left hemisphere—frontal lobe (AF3, AF7, F3, F5, F7), temporal lobe (FT7, T7, TP7), and parietal lobe (CP1, CP3, P1, P3); in the right hemisphere—frontal lobe (AF4, AF8, F4, F6, F8), temporal lobe (FT8, T8, TP8), and parietal lobe (CP2, CP4, P2, P4).

## 4. Results

Given the relatively small sample size in this study and the potential presence of electrophysiological noise in EEG data, prior to formal data analysis, we conducted a normality test on the dataset. The results of the Kolmogorov–Smirnov test revealed non-normal distributions in the high-frequency alpha power data for both the low novelty and high humor groups (*p* = 0.013, *p* = 0.008). Consequently, we performed a normalization transformation of these two sets of data to ensure the accuracy and reliability of the results.

### 4.1. Behavioral Results

**Figure 2:**
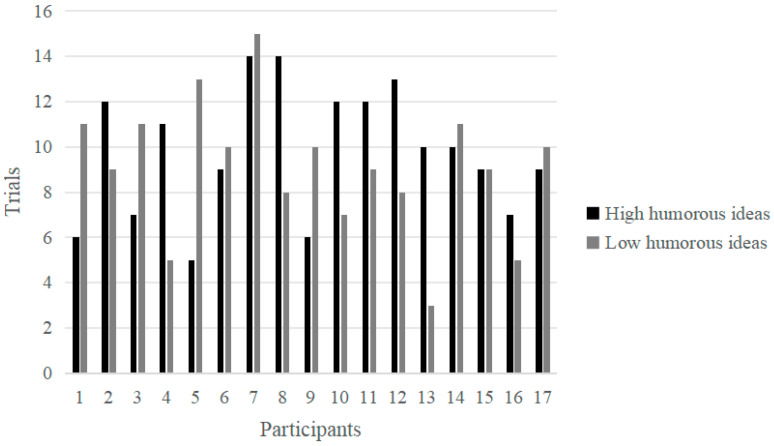
The trials of self-evaluated high and low humorous responses in the humor group.

**Figure 3:**
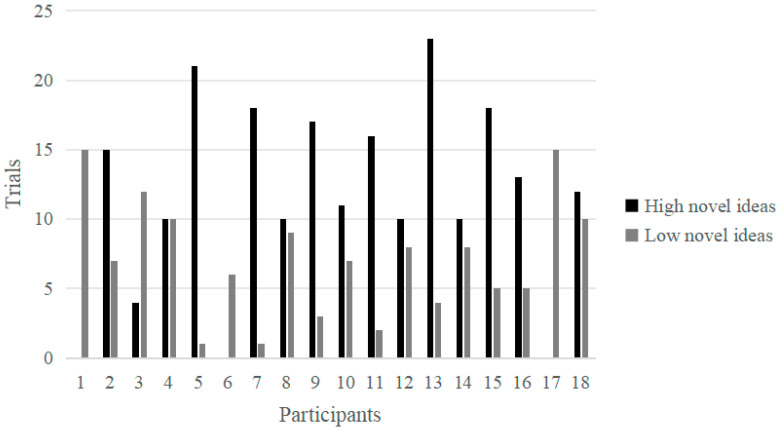
The trials of self-evaluated high and low novel responses in the novelty group.

Based on the humor self-evaluation scores, the first 33% (more than 5 points) and the last 33% (less than 3 points) were identified across all participants as the high and low score groups, respectively, with 166 and 154 effective trials. Similarly, for the novelty self-evaluation scores, the first 33% (more than 5 points) and the last 33% (less than 3 points) were identified as the high and low score groups, respectively, with 208 and 128 effective trials. The trials in which every participant from the humor group self-assessed their responses as either high humor or low humor, along with the trials where all participants from the novelty group self-evaluated their responses as high novelty or low novelty, were illustrated in Figure 2 and Figure 3, respectively.

The average response time for the humor group was 32.80 s, while the average response time for the novelty group was 31.42 s. The response time of participants in the humor group to generate high humorous answers was significantly lower than that of low humorous answers [*F* (1,318) = 54.85, *p* < 0.001, *η_p_*^2^ = 0.15]. The response time of participants in the novelty group to generate high novel answers was significantly lower than that of low novel answers [*F* (1,334) = 29.300, *p* < 0.001, *η_p_*^2^ = 0.08]. Additionally, no significant difference was found between the response time of high humor and high novel answers [*F* (1,372) = 1.57, *p* = 0.21]. The specific results can be found in Table 1.

Furthermore, gender differences in response times were observed between the humor and novelty groups. More specifically, among participants in the novelty group, males (*M* = 36.79, *SD* = 9.24) exhibited response times that were marginally higher than their female counterparts (*M* = 28.00, *SD* = 9.99) [*t*(16) = 1.87, *p* = 0.08]. In the humor group, male response times (*M* = 39.64, *SD* = 10.95) were significantly higher than those of females (*M* = 27.33, *SD* = 11.78) [*t*(15) = 2.27, *p* < 0.05]. However, there were no statistically significant gender differences in the levels of humor [*t*(15) = 0.11, *p* = 0.91] and novelty [*t*(16) = 0.96, *p* = 0.35] observed in the viewpoints generated by participants in both the novelty and humor groups. 

### 4.2. EEG Results

In this section, we included the top 33% of answers categorized as either humorous or novel in the statistical analysis. We conducted a 2 (answer type: high humorous, high novel) × 3 (time period: early, middle, and late) × 2 (hemisphere: left hemisphere, right hemisphere) repeated measures ANOVA. 

#### 4.2.1. The Differential Power of Humor Generation and Creative Generation in Low Alpha Frequency Bands

**Figure 4:**
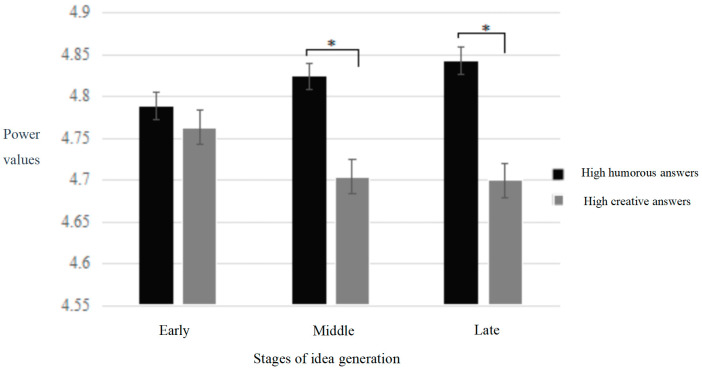
Temporal changes in low-frequency alpha power in the frontal area during humor generation and creative generation. Note: “*” denotes a significance level of 95%, the same applies below.

In the frontal area, the results revealed a significant interaction effect of answer type and time period in the frontal area [*F* (2,68) = 3.49, *p* < 0.05, *η_p_*^2^ = 0.11]. Simple effect analysis indicated that the power value of humorous answers was significantly higher than that of novel answers in the middle (*p* < 0.05) and late (*p* < 0.05) stages (refer to Figure 4). In the temporal area, a significant main effect of time period was observed [*F* (1,34) = 5.80, *p* < 0.01, *η_p_*^2^ = 0.18], with subsequent multiple comparisons revealing that the power value in the early stage was significantly higher than that in the middle and late stages. In the parietal area, no significant results were found. 

#### 4.2.2. The Differential Power of Humor Generation and Creative Generation in High Alpha Frequency Bands

**Figure 5:**
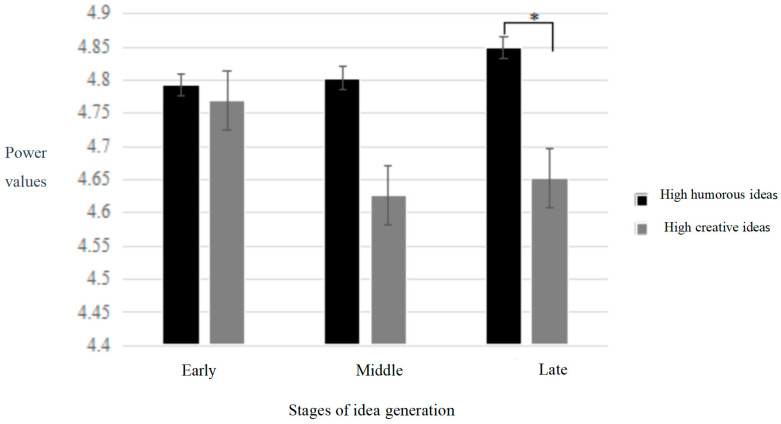
Temporal changes in high-frequency alpha power in the right frontal area during humor generation and creative generation.

In the frontal area, we observed a marginally significant triple interaction effect involving answer type, time period, and hemisphere [*F* (2,34) = 2.81, *p* = 0.069, *η_p_*^2^ = 0.09]. Further simple effect analysis revealed that in the late stage, the power value of high humorous answers was significantly higher than that of high novel answers in the right frontal area (*p* < 0.05) (refer to Figure 5). In the temporal area, a significant main effect of time period was identified [*F* (2,34) = 8.37, *p* < 0.005, *η_p_*^2^ = 0.24], with the power value in the early stage significantly higher than that in the middle and late stages. In the parietal area, a significant main effect of hemisphere was found [*F* (1,34) = 7.36, *p* < 0.05, *η_p_*^2^ = 0.21], with the power value of the right parietal region being significantly higher than that of the left parietal region.

## 5. Discussion

### 5.1. Insight Characteristics of Humor Generation and Creative Generation

Behavioral findings indicate that generating humorous or novel answers takes less time than generating non-humorous or non-novel answers, suggesting that increased thinking time does not necessarily correlate with higher levels of humor or novelty. This suggests that the process of generating humorous and novel ideas may possess elements of insight. Kozbelt and Nishioka, in their discussion on the relationship between humor generation and creativity, unified humor generation and creative problem-solving as ill-defined problems [23]. They pointed out that although the underlying mechanisms connecting humor generation and creative problem-solving are not yet clear, both may involve processes related to insight problem-solving. This aligns with the results supported by our study. Simultaneously, EEG data reveals that both humor generation and creative generation exhibit significantly higher power values in the right parietal area compared to the left. Previous studies have confirmed elevated alpha power in the right parietal and temporal regions before insight problem solving [41,42]. Moreover, generating insightful solutions, as opposed to simply recognizing answers based on prompts, is associated with an increase in alpha power in the right parietal lobe [43]. 

Taken together, these findings suggest that the cognitive processes involved in humor generation and creative generation may share some characteristics with insight. This may be attributed to the daily conversation materials used in the experiment, requiring individuals to suppress conventional ideas induced by the context and interpret specific content provided by the context from a new perspective. Consequently, the shift from conventional ideas to novel ideas inherent in humor generation and creative generation bears some resemblance to the process of “breaking the mindset” in insight problem solving.

### 5.2. Comparisons of Humor Generation and Creative Generation across Early, Middle, and Late Stages

#### 5.2.1. The Early Stage

From a temporal perspective, both in the low-frequency alpha and high-frequency alpha, a main effect of time period was observed in the temporal region. Specifically, the power of low-frequency alpha and high-frequency alpha during the early stage is significantly higher than that in the middle and late stages. The temporal region is closely linked to semantic activation [36] with the right anterior temporal area and is particularly associated with the remote connection of information during reading and comprehension [41]. In our study, participants were asked to read the provided context information, and the heightened alpha power in the temporal area during the early stage may reflect the activation and retrieval of extensive semantic information as participants engage with the context.

The findings from the early stage also suggest that humor generation and creative generation may share similar cognitive processing during the initial phases of idea generation. Researchers have observed significant conceptual similarities between humor and creativity, both necessitating the novel association of disparate elements to evoke surprise or pleasure [44]. Humorous individuals are often deemed creative, as they connect seemingly unrelated concepts in a unique manner to entertain others. Consequently, humor generation is considered an expression of creative potential within the realm of humor [24].

#### 5.2.2. The Middle and Late Stages

In the middle and late stages, we found that the low-frequency alpha oscillation of humorous ideas in the frontal region was significantly higher than that of novel ideas. The frontal lobe, particularly the inferior frontal gyrus, is associated with idea selection [45,46]. Low-frequency alpha activation is associated with a decrease in cerebral cortex activity, which is characterized by a temporary inhibition of executive function and logical thinking processes [27], sometimes described as cortical idling [47] or intuitive processing [48]. Therefore, the higher low-frequency alpha oscillation of humorous ideas in the frontal region in the middle and late stages may reflect the role of the emotional goal of humor in the idea selection stage. Specifically, different from creative generation, when engaging in humor generation, individuals automatically establish an emotional goal before the contextual presentation. This entails generating answers capable of eliciting joyous emotional experiences or laughter, aligning with the task requirements. During the early stages of humor generation, individuals employ thinking strategies akin to those in creative generation, such as retrieving information from long-term memory, engaging in mental imagery, or adopting perspective shifts. However, during the selection of humorous semantic content, influenced by the emotional aim of humor, individuals do not favor viewpoints generated through rigorous logical reasoning, as opposed to creative generation. On the contrary, they tend to opt for so-called “twisted” or even self-contradictory perspectives. This preference aligns with the ultimate goal of humor, which aims to achieve emotional communication by eliciting joyous emotional experiences or laughter. Minsky viewed humor as an emotional form of thought processing, the elimination of logical problem solving [49], providing some degree of support for the role of emotional aims.

Additionally, in the late stage, the high-frequency alpha power oscillation of humorous ideas in the right frontal region was significantly higher than that of novel ideas. High-frequency alpha is usually associated with specific task needs [50,51], reflecting top-down active inhibition in brain activity. To generate humorous ideas, participants need to suppress not only semantically irrelevant or ordinary ideas but also novel yet non-humorous ideas when selecting humorous ideas. Consequently, the high-frequency alpha oscillation level in the right frontal region of humor ideas generation in the late stage is significantly higher than that of novel ideas, which may reflect the inhibition process of novel ideas in the process of humor ideas selection. The findings from the middle and late stages also suggest potential cognitive processing differences between humor generation and creative cognition, possibly stemming from the influence of humor’s emotional objectives.

## 6. Limitations and Future Research

This study, based on a comparative perspective of humor and creativity, revealed for the first time the shared and distinctive neural mechanisms in active humor generation and creativity, which hold theoretical significance and practical value. However, considering the prevailing neglect of cognitive research on humor generation by researchers, there remains a substantial journey ahead in exploring the cognitive intricacies of humor generation in future studies.

First, due to limitations in experimental technical conditions, achieving precise brain area localization for specific cognitive processes in humor generation is challenging in this study. As known, although EEG provides high temporal resolution, its spatial resolution is relatively low. In future research, researchers may consider utilizing functional magnetic resonance imaging (fMRI) as an alternative while acknowledging the ecological validity issues associated with the spatial constraints of fMRI technology in experimental settings. Second, building upon the exploration of the psychological processes involved in humor generation, there is a continued focus on potential predictor variables that may influence humor generation, especially factors that might impact different stages of humor generation. In the realm of research on creative cognition, Mumford and colleagues found that various academic disciplines emphasized different cognitive processes in creative problem solving [52]. For instance, social sciences highlight concept combination and viewpoint generation, while biological sciences underscore information gathering and idea evaluation. Similarly, humor generation is highly contextual, implying that when investigating humor creation, it is crucial to elucidate whether there is a phenomenon of emphasis on specific cognitive processes under different contexts. Third, by focusing on the specific cognitive processes involved in humor generation, intervention programs can be developed to enhance humor generation abilities. Scott et al. highlighted that the most effective creativity training programs are those designed to foster key creative thinking processes through continuous teaching and exercises [53]. Future researchers may explore the extension of intervention approaches and strategies aimed at improving creativity to the realm of enhancing humor abilities. Simultaneously, by delving into the specific cognitive processing involved in humor generation, a more comprehensive and reliable set of intervention measures for enhancing humor abilities can be formulated. Third, certain individual-level variables, including aspects like humor appreciation, creative skills, and personality, have the potential to impact the psychological processes involved in humor generation [19,20]. Unfortunately, the study has overlooked variability at the individual level. Subsequent future research could further explore individuals exhibiting high and low humor sensitivity, aiming to uncover shared and distinctive psychological processes underlying humor generation. Additionally, examining the key distinctions in the humor generation process for individuals with varying levels of humor sensitivity would be valuable.

## 7. Conclusions

This study delved into the neurophysiological underpinnings of humor generation by drawing comparisons with creative generation. The findings suggested that in the early stages of viewpoint generation, both humor and creative generation exhibited similar processing patterns involving the activation and retrieval of long-term memory information based on contextual cues. The distinctions between the two primarily manifest in the middle and late stages, particularly in the integration and selection of ideas, where the selection of humorous ideas required inhibiting not only irrelevant or ordinary ideas, akin to creative generation but also novel yet non-humorous ideas. 

## Figures and Tables

**Table 1:** The response time of high/low humor group and high/low novelty group.

	*M*	*SD*	*F*	*p*
High humor group	24.8	0.21	54.85	<0.001
Low humor group	47.0	0.22		
High novelty group	27.5	1.95	29.30	<0.001
Low novelty group	34.5	2.68		

Note: The unit of response time is seconds.

## Data Availability

The data that support the findings of this study are available from the corresponding author or the first author upon reasonable request.
